# Identification of *Pneumocystis jirovecii* with Fluorescence In-Situ Hybridization (FISH) in Patient Samples—A Proof-of-Principle

**DOI:** 10.3390/jof8010013

**Published:** 2021-12-25

**Authors:** Débora Raysa Teixeira de Sousa, João Ricardo da Silva Neto, Roberto Moreira da Silva, Kátia Santana Cruz, Sven Poppert, Hagen Frickmann, João Vicente Braga Souza

**Affiliations:** 1 Mycology Laboratory, Dr. Heitor Vieira Dourado Tropical Medicine Foundation, Manaus 69040-000, Brazil; Debora-raysa@hotmail.com (D.R.T.d.S.); netto.23@hotmail.com (J.R.d.S.N.); robertomsjr@hotmail.com (R.M.d.S.J.); katia.cruz@fmt.am.gov.br (K.S.C.); 2 Diagnostic Department, Bernhard Nocht Institute for Tropical Medicine Hamburg, 20359 Hamburg, Germany; 3 Department of Microbiology and Hospital Hygiene, Bundeswehr Hospital Hamburg, 20359 Hamburg, Germany; 4 Department of Medical Microbiology, Virology and Hygiene, University Medicine Rostock, 18057 Rostock, Germany; 5 Mycology Laboratory, National Institute for Amazonian Research (INPA), Manaus 69067-375, Brazil

**Keywords:** paraffin-embedded tissue, FISH, *Pneumocystis jirovecii*, diagnosis, test evaluation, Grocott’s staining, real-time PCR, bronchoalveolar lavage, sputum

## Abstract

In resource-limited settings, where pneumocystosis in immunocompromised patients is infrequently observed, cost-efficient, reliable, and sensitive approaches for the diagnostic identification of *Pneumocystis jirovecii* in human tissue samples are desirable. Here, an in-house fluorescence in situ hybridization assay was comparatively evaluated against Grocott’s staining as a reference standard with 30 paraffin-embedded tissue samples as well as against in-house real-time PCR with 30 respiratory secretions from immunocompromised patients with clinical suspicion of pneumocystosis. All pneumocystosis patients included in the study suffered from HIV/AIDS. Compared with Grocott’s staining as the reference standard, sensitivity of the FISH assay was 100% (13/13), specificity was 41% (7/17), and the overall concordance was 66.7% with tissue samples. With respiratory specimens, sensitivity was 83.3% (10/12), specificity was 100% (18/18), and the overall concordance was 93.3% as compared with real-time PCR. It remained unresolved to which proportions sensitivity limitations of Grocott’s staining or autofluorescence phenomena affecting the FISH assay accounted for the recorded reduced specificity with the tissue samples. The assessment confirmed *Pneumocystis* FISH in lung tissue as a highly sensitive screening approach; however, dissatisfying specificity in paraffin-embedded biopsies calls for confirmatory testing with other techniques in case of positive FISH screening results. In respiratory secretions, acceptable sensitivity and excellent specificity were demonstrated for the diagnostic application of the *P. jirovecii*-specific FISH assay.

## 1. Introduction

Pneumocystosis is a life-threatening opportunistic infection that affects immunocompromised patients, especially those within the acquired immunodeficiency syndrome (AIDS) stage of the human immunodeficiency virus (HIV) infection, but also those with other immunocompromising conditions like haematological malignancies, inflammatory disease, and steroid therapy [1,2]. Due to a particularly high risk for patients with CD-4 lymphocyte counts < 200/µL, trimethoprim-sulfamethoxazole (TMP/SMX) prophylaxis is recommended for those individuals [3]. TMP/SMX is also the therapeutic drug of first choice [4].

Focusing on the aetiology of the disease, pneumocystosis is caused by the fungal pathogen *Pneumocystis (P.) jirovecii*, an ascomycete that has an affinity for the pulmonary pathways, which adheres to the wall of the pneumocytes and induces infection there [5,6]. This organism is characterized by not being cultivable under diagnostic routine conditions, a fact that makes its detection and identification difficult [7], so alternative diagnostic approaches [8] including indirect methods like (1-3)-β-D-glucan detection [9] have been suggested.

In short, the diagnosis of pneumocystosis is essentially based on known host factors, in particular immunosuppression [1,2], next to clinical symptoms, radiological images, biochemical and antigenic parameters, as well as on histopathological stains including immunostaining [8,9]. Due to the resulting complex diagnostic algorithms which are based on composite reference standards rather than on individual diagnostic results in a defined diagnostic specimen, it is important to stress that it is challenging to virtually impossible to definitely conclude on the presence or absence of *P. jirovecii* just based on an individual analysed sample material.

To facilitate the diagnosis of other fungal diseases, thorough evaluations of the molecular tool fluorescence in situ hybridization (FISH) for the detection and identification of pathogenic fungi in samples from patients with suspected fungal infections were conducted. Several studies and reviews suggested a considerable potential of this method as an important add-on for a fast, sensitive, and specific diagnosis of fungal diseases [10,11,12,13,14,15,16,17,18,19,20,21,22,23,24,25,26,27,28,29,30,31]. Early diagnosis and specific treatment of fungal infections is fundamental to improve the prognosis of patients, or even to avoid the mortality caused by such infections. In a recent study, FISH was tested in hospitalized patients with candidiasis, leading to considerable improvement in the diagnosis and specific treatment of this infection. The time of identification, time of treatment with the specific antifungal drug, length of stay of the patients in hospital, mortality, and cost per patient including diagnosis and treatment were analysed. With the implementation of FISH, it was observed that the identification time took four hours, while the conventional method required four days. Considering this, there were savings per patient of $415 and decreased mortality [21].

In the present study, the use of an in-house *P. jirovecii*-specific FISH probe was evaluated by performing tests with paraffin-embedded samples containing lung biopsies with positive and negative results for pneumocystosis obtained by conventional methods used in routine. Further, evaluation of *P. jirovecii* FISH was conducted with respiratory secretions like sputum and bronchoalveolar lavage with real-time PCR as the reference standard. By doing so, it was assessed whether FISH may also contribute to facilitating routine diagnosis of *P. jirovecii*. Of note, the included pneumocystosis patients suffered from HIV/AIDS as the underlying disease.

## 2. Materials and Methods

### 2.1. Paraffin-Embedded Tissue Samples

The *P. jirovecii*-specific fluorescence in situ hybridization (FISH) assay was evaluated with paraffin blocks containing lung biopsies from patients diagnosed with HIV/AIDS and pneumocystosis as cause of death and also from patients with other underlying pulmonary diseases. All samples were taken at the Fundação de Medicina Tropical Heitor Vieira Dourado (FMT-HVD) between 1996 and 2013. In total, the sample set comprised 13 histologically confirmed cases of pneumocystosis as causes of death of the patients next to 17 cases who had other fatal pulmonary conditions. For the latter population, HIV status was not documented. Only tissue blocks comprising post-mortally taken lung biopsies were included in the assessment. In order to prepare them for FISH and histological analyses, the blocks were cut in a microtome (MicroTec 4050/USA) to obtain successive, thin, uniform cuts of approximately 5 to 7 microns. After that, the sections were fixed on slides pre-treated with polylisin and labelled with the information on the year of sampling as well as on the necropsy number. After all the slides were ready for further sample processing, neighboring slides of the ones chosen for FISH were subjected to Gomori Grocott’s staining, which is a specific histological staining technique for fungi including *Pneumocystis* spp. [32]. In this study, Grocott’s staining [33] was performed as a reference standard in order to define positive and negative samples. Prior to either FISH or Grocott’s staining, all slides were deparaffinized first in two xylene immersions lasting 10 min each. Subsequently, deparaffinization was proceeded by immersions in alcohol sequences with different concentrations (100%, 80%, and 70%) for 10 min each, after which the slides were subjected to drying at room temperature.

### 2.2. Deep Airway Secretions

In order to evaluate the *P. jirovecii* probe for FISH with respiratory secretions, a total of 30 samples including sputum (*n* = 17, no hints for sputum induction on the sample accompanying sheets) and bronchoalveolar lavage (BAL, *n* = 13) were obtained from HIV/AIDS patients admitted to the FMT-HVD presenting symptoms suggestive of pneumocystosis between October 2015 and June 2016. Details on the symptoms of the HIV/AIDS patients leading to the clinical suspicion of pneumocystosis were not specifically reported to the laboratory but usually comprise radiological signs of interstitial pneumonia and suggestive laboratory findings like increased lactate dehydrogenase levels in severely immunosuppressed patients [8,9]. After arrival at the mycology laboratory of this institution, the specimens were centrifuged at 2000 rpm (rounds per minute) for 10 min using an Excelsa Baby II 206-R Ind. Brasileira centrifuge (Fanem, Cumbica, Guarulhos, Brazil). Subsequently, the samples were smeared on slides for microscopical assessment, resulting in circular smears of approximately 1.5 cm in diameter. Afterwards, the slides were dried at room temperature and immersed in sequences of alcohol at descending concentrations (100%, 80%, and 70%) for 10 min each. Then, the slides were once more dried at room temperature prior to further FISH assessment.

As a comparative reference standard, the respiratory samples were assessed by in-house real-time PCR for *P. jirovecii* applying a recently described protocol [34]. In detail, the PCR was run as a Sybr Green-based assay using the forward primer 5′-GATCCGAGACATGGTCGCTATT-3′ and the reverse primer 5′-TTCAACCTCCTTCATGGAAACAG-3′ but no hybridization probe in contrast to the described protocol. Melting temperature within the 76.3 °C ± 0.3 °C range was expected in this assay and the amplicons were additionally visualized in agarose gels. In a previous multicentric comparison based on standardized samples [34], the hybridization probe version of the applied assay showed acceptable sensitivity and high specificity, thus confirming its general appropriateness for diagnostic purposes.

### 2.3. In Silico Evaluation of the FISH Probe

The in silico evaluation of the assessed FISH probe was performed with the probeCheck software using the sequence collection SILVA [35,36]. Applying the internet-based software tool “Oligo Calc: Oligonucleotide Properties Calculator” (http://biotools.nubic.northwestern.edu/OligoCalc.html, last accessed on 21 December 2021), melting temperature (Tm), GC content, and potential tendencies towards hairpin formation of the probe were checked. In line with previous recommendations [37,38], a variable region of highly abundant ribosomal RNA with 20 and 30 base pairs length was chosen as the target for the FISH probe; because such probe lengths best combine sensitivity and specificity with easy penetration into the cells.

### 2.4. P. jirovecii FISH-Protocol with Paraffin-Embedded Tissue Samples

The hybridization mix was prepared as follows. In a microtube, 5 ng of the *P. jirovecii*-specific FISH probe PJ1 (5′-GGCTTCATGCCAACAGTCC-3′, conjugated to a Cy5 fluorophore) and of the previously described Cy3-conjugated pan-eucaryotic probe [37] each were added to 10 µL hybridization buffer containing 30% formamide as optimized previously. In detail, the hybridization buffer composition was as follows: 0.9 M NaCl, 0.01% *w*/*v* SDS, 20 mM Tris-HCl pH 7.2, 30% formamide, and 1 µM of probe. For the FISH reaction, the slides were incubated for 2 h at 55 °C in line with the probes’ calculated melting temperature (see “Results” chapter for details). Afterwards, excess probe was removed by immersing the slides for 20 min in washing solution (5 mM Tris, 15 mM NaCl and 0.1% Triton X-100) at 55 °C as described [20]. Subsequently, the slides were counterstained with DAPI (4′6′-diamidino-2-phenylindol), which is specific for nucleic acids. After that, the excess dye was removed again in a washing solution, and finally, the slides were air-dried at room temperature and mounted on a glass slide. In detail, the slides were mounted with Vectashield solution (Vector, Burlingame, CA, USA) and examined with a Zeiss Axioskop 40 microscope (Zeiss, Jena, Germany) equipped with a standard filter set as detailed elsewhere [39].

### 2.5. P. jirovecii FISH Protocol with Deep Airway Secretions

The FISH assay was performed as described by [40]. Slides containing fixed sample materials after preparation as described above were overlain with probe-containing hybridization buffer (0.9 M NaCl, 0.01% *w*/*v* SDS, 20 mM Tris-HCl, pH 7.2, 30% formamide, and 1 µM Cy3-labeled *P. jirovecii*-specific FISH probe PJ1 probe) and incubated at 55 °C for 2 h. After this period, the slides were washed using washing buffer (20 mM Tris-HCl, pH 8.0, 0.01% *w*/*v* SDS, 5 mM EDTA, and 150 mM NaCl) for 20 min. In addition to the specific staining with the probe, all samples were counterstained with DAPI (4′,6-diamidino-2-phenylindole-dihydrochloride). The stained slides were dried at 37 °C for 20 min and afterwards examined with a Zeiss Axioskop 40 microscope (Zeiss, Jena, Germany).

### 2.6. Ethics

Ethical clearance for the anonymized use of the sample materials for the applied test comparison was provided by the Human Research Ethics Committee of the Dr. Heitor Vieira Dourado Tropical Medicine Foundation without requirement for informed consent. The reference numbers for the ethical clearances were 50817615.2.0000.0005 for the assessment of the respiratory samples and 23715813.80000.0005 for the assessments of the post-mortally taken lung biopsies. The study was performed in line with requirements of the Declaration of Helsinki and all its amendments.

## 3. Results

### 3.1. In Silico Evaluation

In silico evaluation of the applied FISH probe PJ1 indicated 100% matching with the 18S rRNA gene of *P. jirovecii* (GenBank accession number AB266392). The also observed perfect matching with respective sequences of the species *P. murina* (GenBank accession number AY532651), *P. wakefieldiae* (GenBank accession number L27658), and *P. carinii* (GenBank accession numbers S83267 and X12708) were considered as clinically irrelevant due to the pronounced host-specificity of *Pneumocystis* spp. [4,7]. Assessment with the internet-based software tool “Oligo Calc: Oligonucleotide Properties Calculator” (http://biotools.nubic.northwestern.edu/OligoCalc.html, last accessed on 21 December 2021) indicated a 58% GC-proportion as well as a salt-adjusted melting temperature of 60 °C and excluded potential hairpin formation in silico.

### 3.2. In Vitro Evaluation with Paraffin-Embedded Tissue Samples

After in-silico evaluation, validation of *P. jirovecii* FISH with paraffin-embedded tissue samples was performed as follows. In order to detect *P. jirovecii* in paraffin-embedded tissue blocks containing lung biopsies, FISH reactions applying the PJ1 probe were compared to traditional histological staining. The latter was applied as the reference method in this assessment, defining the positive and negative controls for the evaluation. In detail, histological sections of the same block were investigated using Grocott’s staining.

**Figure 1:**
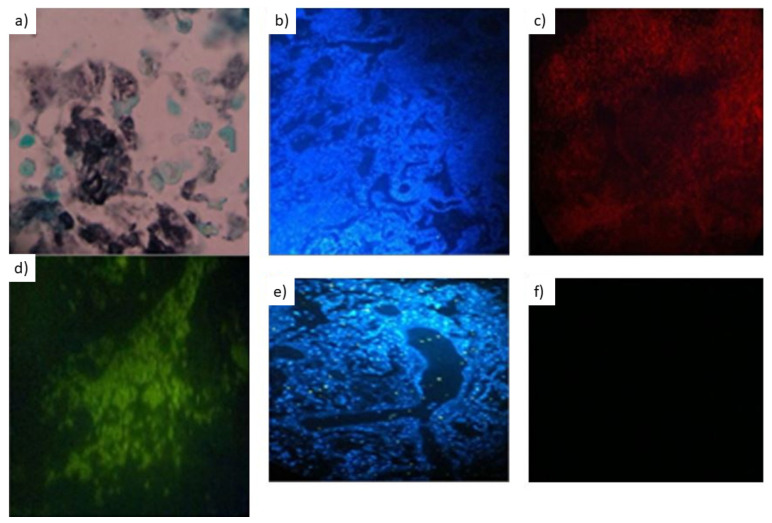
FISH staining results in paraffin-embedded tissue applying paneukayotic and *P. jirovecii-specific* FISH probe. Magnification 1000×. No size band available, so the 5–8 µm size of the *Pneumocystis* cysts serves as a size standard. The positive sample row—(**a**–**d**)—shows the staining with (**a**) Grocott’s stain (counterstained with Goldner’s stain III), (**b**) the DAPI dye visualized in blue, (**c**) the pan-eukaryotic probe visualized in red, and (**d**) the *P. jirovecii* probe visualized in green, respectively. Subsequently, the negative sample row—(**e**,**f**)—shows the staining (**e**) with the DAPI dye and (**f**) the *P. jirovecii* probe, respectively. On note, only cysts were seen.

As indicated in Table 1, which is the cross-table indicating co-positivity and co-negativity of FISH as competitor approach and histopathology applying Grocott’s staining as reference standard for the detection of *P. jirovecii* in paraffin-embedded lung tissue (Figure 1), the FISH assay applying the PJ1 probe achieved 100% sensitivity and 41% specificity with an overall concordance of 66.7% (Table 1).

### 3.3. In Vitro Evaluation with Respiratory Secretions

**Figure 2:**
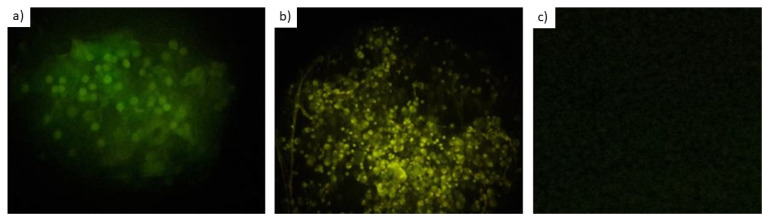
FISH staining results in respiratory secretions with sputum and BAL (bronchoalveolar lavage) samples. Magnification 1000×. No size band available, so the 5–8 µm size of the *Pneumocystis* cysts serves as a size standard. (**a**) Positive sputum sample, stained with the probe JP1 targeting *P. jirovecii*, visualized in green. (**b**) Positive BAL sample, stained with the probe JP1 targeting *P. jirovecii*, visualized in green. (**c**) Negative sputum sample. Of note, Grocott’s staining was not applied with respiratory samples in this study and only cysts were seen.

For the evaluation of *P. jirovecii* FISH with 30 respiratory secretions obtained from HIV-/AIDS patients (Figure 2) with suspicion of *P. jirovecii* infections, in house real-time PCR was used as the diagnostic reference standard. For all PCR-positive samples, the recorded cycle threshold (Ct) values were in a robust 30.6 ± 0.6 cycle range with melting curves in the expected temperature spectrum as well as with visible bands in agarose gels. A positive result was never based on the amplification curve and the Ct value alone, because non-specific SybrGreen signals were observed at a mean Ct value of 34.8. So, assessments of melting curves and agarose gels were always included in the diagnostic interpretation. Compared to real-time PCR, sensitivity of FISH was 83.3% with 100% specificity and 93.3% overall concordance of real-time PCR and FISH. Details are provided in Table 2.

**Figure 3:**
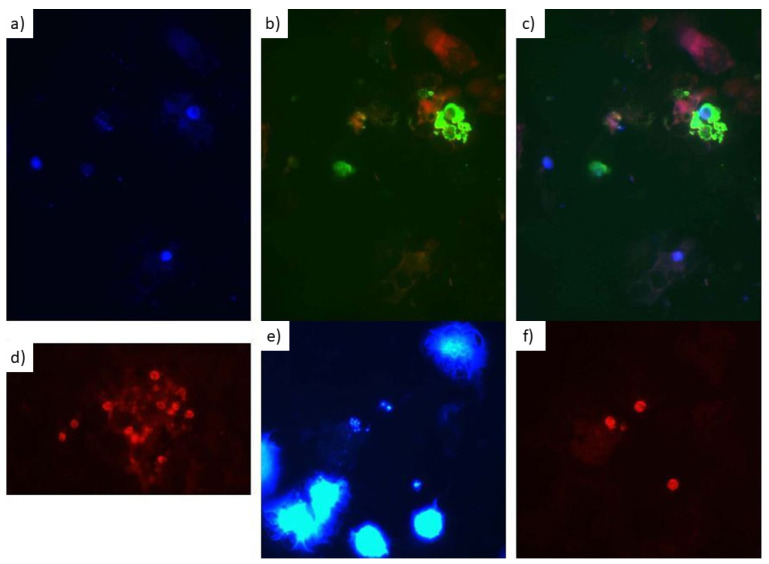
FISH staining results with and without counterstaining. Magnification 1000×. No size band available, so the 5–8 µm size of the *Pneumocystis* cysts serves as a size standard. The figure components (**a**–**c**) show the identical sample with (**a**) calcofluor fluorescent staining visualized in blue, (**b**) *P. jirovecii* FISH staining with a fluorescein-labelled probe visualized in green and (**c**) an overlay of both staining approaches in order to show the matching. (**d**) *P. jirovecii* FISH alone with a probe labelled with the cyanine fluorophore Cy3 visualized in red. The figure components (**e**,**f**) show the identical sample with (**e**) staining with the DNA stain DAPI visualized in blue and (**f**) *P. jirovecii* FISH staining with a Cy3-labelled probe visualized in red. The corresponding position of *P. jirovecii* DNA and *P. jirovecii*-specific FISH-staining of the cells’ cytoplasm suggest specific staining and not mere autofluorescence. Of note, only cysts were seen.

Of note, the degree of autofluorescence as well as the contrast of the microscopic images obtained varied depending on the fluorophore applied. Thereby, the cyanine fluorophore Cy3 provided clearer results with less autofluorescence compared with 6-carboxyfluoresceine (6-FAM) (Figure 3).

## 4. Discussion

The study confirmed the suitability of fluorescence in situ hybridization (FISH) for the identification of *P. jirovecii* both in paraffin-embedded tissue and in respiratory secretions as a rapid, easy-to-perform, and cost-efficient diagnostic procedure for resource-limited tropical endemicity settings. With focus on both types of sample materials and the respective applied reference methods, however, the results call for a more differentiated discussion.

Although the study successfully provided a proof-of-principle for the sensitive FISH-based detection of *P. jirovecii* in lung tissue, the dissatisfying specificity of the assay with paraffin-embedded tissue is bothersome and requires further interpretation. As known from previous assessments, the distribution of *Pneumocystis* cells in different slices may have been uneven as observed for other eucaryotic pathogens in tissue samples [40,41,42]. Second, various factors affect the visibility of target structures in histological slides [43]. Third, the diagnostic accuracy of Grocott’s staining for the identification of *P. jirovecii* is imperfect as repeatedly shown [32,44,45,46,47,48,49,50,51,52]. In detail, both sensitivity and specificity of Grocott’s staining for the diagnostic identification of *P. jirovecii* is very different from 100%. In a veterinary medical study comparing conventional in situ hybridization (ISH) with Grocott’s staining in lung tissue specimens of pigs, Grocott’s staining had missed 12/32 (37.5%) *Pneumocystis* spp.-positive samples [53], challenging its suitability as a reference standard or even a “gold standard”. For the latter, perfect diagnostic accuracy is assumed by definition [54], a condition that can rarely be fulfilled by any diagnostic assay.

On the other hand, it remains uncertain whether sensitivity problems of Grocott’s staining alone may have accounted for a FISH specificity of as low as 41% as compared with the reference standard. Therefore, it is an undeniable limitation of the study that alternative diagnostic approaches other than Grocott’s staining like antibody-staining [44,47] as well as modern molecular approaches [55,56] or flow-cytometry from concomitantly taken respiratory samples [57] were not applied to support the decision on the “true” *P. jirovecii* infection state of each assessed sample, as specimens different from paraffin-embedded tissues had not been taken during the autopsies. Unfortunately, the real-time PCR assay for *P. jirovecii*, which was applied with the assessed respiratory secretions taken from other patients in this study, was not evaluated with paraffin-embedded tissues. As formalin-fixed, paraffin embedded tissues are, however, pretty tough challenges for pathogen-specific nucleic acid amplification techniques due to nucleic acid alterations like, e.g., formalin-induced deamination of cytosine to uracil, single strand breaks, etc. as extensively discussed elsewhere [40,41,58,59,60,61], we abstained from applying the Sybr Green PCR approach with such difficult specimens.

Autofluorescence, which is a considerable challenge for FISH with more complex sample matrices as summarized recently [14], may be an alternative explanation for the recorded poor specificity. Admittedly, tissue specimens are pretty tough materials for FISH regarding a variety of interfering factors affecting the diagnostic accuracy as detailed elsewhere [62]. To the authors’ experience, visualization of staining with the FITC/FAM (fluorescein isothiocyanate/fluorescein amidite) dyes is particularly prone to autofluorescence, while this phenomenon is less pronounced e.g., with cyanine dyes like the fluorophores Cy3 and Cy5 which were applied with the paraffin-embedded tissues. Unfortunately, potentially non-specific reactions in the tissue samples were observed in spite of the use of the Cy5 channel. As also experienced by the authors in previous attempts (unpublished data), sample preparation with osmium tetroxide, which has even been discussed for use as a weapon due to its very high toxicity [63], may reduce the autofluorescence phenomenon at least in respiratory samples. This procedure, however, was not attempted with the tissue samples, because the substance was considered as too toxic for use in diagnostic routine settings. Further, nonsense probes like NON EUB338 [14,64] may be applied to at least control autofluorescence in FISH, an approach which was not chosen in this study but should be considered in case of diagnostic applications of the described FISH assay.

Interestingly, however, no non-specific *P. jirovecii* FISH signals were observed within the respiratory samples, which were processed as suggested previously [65]. This observation does not exclude autofluorescence in the tissue samples by itself, as both the procedures of the sample preparation as well as the sample types themselves were different, but further reduces its likeliness. So, the excellent specificity of *P. jirovecii* FISH in respiratory specimens makes apparently reduced specificity of FISH in paraffin-embedded tissue just due to insufficient sensitivity of Grocott’s staining even more likely.

Compared to in-house real-time PCR, sensitivity of *P. jirovecii* FISH from respiratory secretions was slightly reduced. This is not surprising, as high sensitivity of the applied in-house real-time PCR is known from a previous multicentric test comparison study [34], while FISH has similar detection limits as other microscopical approaches [14]. Of note, the very high concordance of real-time PCR and FISH results together with the low overall sample count made a meaningful comparison of diagnostic accuracy in bronchoalveolar lavage and sputum unfeasible. Unfortunately, Grocott’s staining from the respiratory samples had not been performed. Otherwise, differences in the sensitivity of Grocott’s staining and of FISH for the detection of *P. jirovecii* in respiratory samples might have been assessed in comparison to real-time PCR as well. Of note, however, previous assessments have even suggested a potentially too high sensitivity of *P. jirovecii*-specific nucleic acid amplification assays from the clinical point of view, making the discrimination of clinically apparent infection and harmless colonization challenging. In more detail, high rates of *P. jirovecii* colonization are well known from autopsy materials from the lungs of deceased individuals without *P. jirovecii*-associated pneumonia and even without signs of pulmonary inflammation [66,67,68], so the clinical interpretation of positive *P. jirovecii* PCR-results remains an issue of ongoing academic debate [68,69] and should always consider other clinical, radiological, and laboratory findings as well.

The study has a number of limitations. First of all, available tissue sample counts for the assessment were limited due to the low frequency of fatal pneumocystosis in spite of collecting tissue samples about several years. Second, the applied reference standard, Grocott’s staining, is associated with limited sensitivity for the diagnosis of *Pneumocystis* spp. in tissue as, e.g., suggested by a previous ISH-based evaluation with pig tissue [53], so it cannot be excluded that the recorded limited specificity of *P. jirovecii* FISH with tissue samples was at least partially due to sensitivity limitations of Grocott’s staining. On the other hand, other specificity data on *Pneumocystis*-FISH do not exist. So, due to the lacking application of another reference standard, the study results allow the demonstration of the specificity of *Pneumocystis* FISH in comparison to Grocott’s staining only but they do not allow a final evaluation of the true specificity of *Pneumocystis* FISH in tissue. Third, additional diagnostic approaches to confirm or exclude the abundance of *P. jirovecii* within the paraffin-embedded tissue samples were not conducted. Fourth, attempts to further reduce potential autofluorescence within the samples applying toxic chemicals were not performed the same as the control of autofluorescence by the application of nonsense FISH probes. Fifth, different reference standards were applied for the assessment with paraffin-embedded tissue and the assessment with respiratory secretions, making the results of both subgroup analyses within the study not directly comparable. Sixth, due to funding constraints for the study, the real-time PCR assay had to be run as a less expensive Sybr Green-based approach instead of using a hybridization probe like in its evaluation study [34], so it can only be speculated whether its test characteristics were still the same with this modification. Seventh, only very restricted clinical information was available to our laboratory, making the interpretation regarding the individual etiological relevance of detected *P. jirovecii* cysts and pathogen DNA challenging. A small range of measured cycle threshold values of PCR-positive samples and lacking absolute quantification in real-time PCR did not allow a quantification-based estimation of etiological relevance, too. Eighth, only pneumocystosis patients with HIV/AIDS as underlying disease were included, which limits the general interpretability of the diagnostic accuracy due to reported differing pathogen loads in non-HIV-infected pneumocystosis patients [1,2]. Ninth, resource limitations affecting the quality of the available microscopy and photography equipment resulted in very limited quality of the photo documentation for this study. On the other hand, the robust FISH approach allowed diagnostic interpretation in spite of the use of simple microscopic equipment, which makes the assay suitable for application in resource-limited tropical settings. Tenth, alternative innovative molecular diagnostic approaches like the RNAscope technique were not included in the assessment, so broader methodical evaluations should be considered for the future.

## 5. Conclusions

In conclusion and in spite of the mentioned limitations, high sensitivity of FISH for the screening for *P. jirovecii* in lung tissue samples and quite acceptable sensitivity in respiratory samples were demonstrated. The recorded dissatisfying specificity of the FISH assay with tissue samples compared with Grocott’s staining, however, suggests confirmatory testing in order to confirm or exclude pneumocystosis in case of positive FISH-based screening results with such sample materials. For example, molecular assessments from respiratory secretions may be considered. Excellent specificity of *P. jirovecii*-FISH with respiratory samples, in contrast, makes the limited sensitivity of Grocott’s staining compared to *P. jirovecii* FISH an alternative explanation for the seemingly poor specificity of the FISH assay with paraffin-embedded tissue samples. Future studies should focus on the clinical interpretation of diagnostic *P. jirovecii* FISH and its positive and negative predictive values for the discrimination of *P. jirovecii*-associated pneumonia in contrast to harmless colonization of the airways with *Pneumocystis* spp. cells. This clinically important topic was beyond the scope of the presented technical evaluation approach.

## Figures and Tables

**Table 1:** Comparison of Grocott’s staining results and FISH staining results with paraffin-embedded tissues. Grocott’s staining was used as the reference standard.

		Grocott’s Staining		
		Positive	Negative	
**FISH**	Positive	13	10	23
	Negative	0	7	7
		13	17	30
**Concordance**				66.7%
**Sensitivity**				100%
**Specificity**				41%

**Table 2:** Comparison of in-house real-time PCR results and FISH staining results with respiratory secretion. Real-time PCR was used as the reference standard.

		In-House Real-Time PCR		
		Positive	Negative	
**FISH**	Positive	10	0	10
	Negative	2	18	20
		12	18	30
**Concordance**				93.3%
**Sensitivity**				83.3%
**Specificity**				100%

## Data Availability

All relevant data are presented with the manuscript text. Raw data can be provided at reasonable request.
